# Infection-induced epigenetic changes and their impact on the pathogenesis of diseases

**DOI:** 10.1007/s00281-020-00793-1

**Published:** 2020-04-21

**Authors:** Nicole Fischer

**Affiliations:** Institute of Medical Microbiology, Virology and Hygiene, University Medical Center Hamburg-Eppendorf, Hamburg, Germany

Epigenetic modifications play a fundamental role in the regulation of cellular gene expression. In addition to their role in mediating short-term responses, some epigenetic marks can also be stably transmitted throughout cell divisions. Such mechanisms are of quintessential importance for the maintenance of cellular identity, and also convey a learning system that allows cells to respond and adapt to environmental changes. In recent years, infections have emerged as one of the triggers, which can profoundly alter epigenetic patterns. For example, the acquisition of immunological memory by the innate system (“trained immunity”) is largely dependent on epigenetic imprinting. Besides their physiological role, it is also becoming increasingly clear that deregulated epigenetic processes contribute to the development of severe diseases, including cancer and auto-inflammatory syndromes. Indeed, an increasing number of studies strongly suggest that infection-associated changes of the cellular epigenome are linked to pathologic processes in acutely or chronically infected organisms. This concept is in particular importance considering chronically persistent infections in which the pathogen manipulates the host cell for its own survival. Given the inheritability of epigenetic changes, another intriguing possibility is that infection-associated epigenetic changes may even alter host cell functions long after the initial infection has been cleared.

Viruses, as well as intracellular bacteria, manipulate their host cell in a way that ensures their maintenance, replication, and transmission. In particular, persistent viruses or chronically infecting bacteria benefit from permanent cellular changes that include regulation of the cell cycle, control of apoptosis, and undermining of the immune system. Some viruses such as Epstein-Barr virus (EBV), Kaposi’s sarcoma-associated herpesvirus (KSHV), or hepatitis B virus use epigenetic mechanisms to ensure the regulation of their genes during chronic or latent infection (a specific form of persistence). In addition to the alteration of the viral genome, viral effector proteins such as the Epstein-Barr virus nuclear antigen (EBNA) proteins of EBV or latency-associated nuclear antigen (LANA) protein of KSHV also interact with cellular chromatin structures [1–4]. For example, the EBNA-3C protein of EBV induces the polycomb-mediated repression of the pro-apoptotic regulatory gene BIM, thus contributing not only to lymphoma development in mice but most likely also to the pathogenesis of human Burkitt lymphoma (BL) [5, 6]. Although changes in epigenetic characteristics have also been described for other persistent viruses (e.g., adenoviruses, polyomaviruses, hepatitis B and C viruses) and intracellular bacteria (*Helicobacter pylori*, chlamydia, mycobacteria, and salmonella), systematic or comparative genome-wide studies on this topic are still largely lacking.

The question of whether such changes persist after the original infection has been cleared has been discussed for a long time but has not yet been able to be directly proven. Despite the lack of direct molecular evidence, there are several indications of the existence of such mechanisms in viral, bacterial, and parasitic infection systems. For example, *Theileria parvum*, a tick-borne parasite transmitted to cattle, causes persistent epigenetic changes in its target cells that persist even after the loss of the parasite [7–9]. A second example is the bacterium *Helicobacter pylori*, a pathogen that is probably the most important risk factor during the multi-stage development of gastric cancer [10–12]. The changes in DNA methylation induced by this pathogen lead to permanent nuclear factor kappa-light-chain-enhancer (NFκB) activation and subsequent chronic inflammation of the gastric mucosa, which persists even after antibiotic eradication of the bacteria.

This review collection on infection-induced epigenetic changes highlights novel insights on how persistent viruses, e.g., herpesviruses, papillomaviruses, hepatitis B virus (HBV), and HIV [13–17], intracellular bacteria (*Mycobacteria*, *Legionella*, *Chlamydia*) [18], and parasites (*Toxoplasma*, *Theileria*, and *Cryptosporium*) [19] hijack host nuclear functions. The chapters describe how pathogens take advantage of the cellular epigenetic network to ensure their replication, persistence, and evasion from the host immune response. The reviews also discuss current knowledge in this newly evolving field how epigenetic changes induced by microorganisms contribute to pathogenesis and memory response in immunity.

This topic is of particular interest in the field of human herpesviruses as these viruses establish a lifelong persistent infection in their host, have co-evolved with their host, and are characterized by biphasic life cycle. While lytic viral replication in producer cells allows the fast reproduction and spread within the host, the latent phase in reservoir cells ensures endurance during cell division and immune evasion. Herpesviruses contain large double-stranded (ds)DNA genomes, which enter the cell as naïve, nucleosome-free DNA. Within the first hours, the viral DNA is decorated with cellular nucleosomes and histone modifications. Within this collection, two human herpesviruses, EBV and KSHV, both associated with tumorigenesis, serve as examples of how chronic persistent viruses use the cellular epigenetic machinery to establish latency, to ensure reactivation and progeny production [14, 16]. In this issue, Buschle and Hammerschmidt focus on the epigenetic regulation importance through the complex life cycle of EBV, while Fröhlich and Grundhoff discuss current evidence of epigenetics in latency establishment and also on how epigenetics and viral protein expression contribute to the pathogenesis of KSHV. KSHV contributes to several malignancies in humans including Kaposi sarcoma (KS), multicentric Castleman’s disease (MCD), and primary effusion lymphoma (PEL). However, KSHV’s molecular contribution to KS and PEL tumorigenesis is not clearly defined. KSHV, different from other human tumor viruses, EBV, and human papillomavirus (HPV), does not encode for a viral protein with the capacity to transform cells by its own. Fröhlich and Grundhoff discuss the hypothesis that tumor cells acquire heritable changes, which in combination with KSHVs’ continued protein expression are responsible for cell transformation [16]. Based on the current knowledge on the interaction of KSHV-encoded proteins and host cell chromatin, the authors speculate on scenarios of how epigenetic alterations might contribute to KSHV-induced cancer.

Similar to EBV and KSHV, high-risk human papillomaviruses (HR-HPyV) are bonafide tumor viruses. HR-HPyV contributes to human cancers of the anogenital and oropharyngeal tract. Different to KSHV and similar to EBV, HPV encodes for viral oncoproteins, called E6 and E7, which (in the case of HR-HPyV) are very potent cellular-transforming proteins by inactivation of cell cycle checkpoints and apoptosis. Burley et al. elegantly describe the unusual life cycle of human papillomavirus, which infects undifferentiated basal epithelial cells of the epidermal layer of the skin [13]. During this initial infection, the viral genome is epigenetically silenced, and these epigenetic modifications change quite dramatically during the differentiation status of the cell resulting in relaxation of viral genome repression and subsequent viral replication. The authors focus on the complex epigenetic regulation of the circular viral genome by histone acetylation mediated by the transcriptional regulator CREB-binding protein (CBP/p300), histone methylation, e.g., polycomb recruitment, and DNA methylation. Thereby, the authors nicely point out the differences in epigenetic changes on the viral genome during productive infection and during pathogenesis and tumorigenesis. Understanding the complex epigenetic regulation of HPV might open new strategies for antiviral treatment in HPV-induced diseases.

Similar to HPV, there is a successful vaccine against HBV. However, chronic HBV infection and its contribution to hepatocellular carcinoma still are a major health burden. HBV can induce a lifelong chronic infection during which the virus persists in the form of a small circular DNA (ccc)DNA in the nucleus of hepatocytes. Epigenetic modifications contribute to the control of viral gene expression mainly by a viral protein, called HBx, and its subsequent recruitment of cellular transcriptional co-activators p300 and histone acetylases (HDAC)1 and Sirtuin1. The review by Dandri provides an encompassing overview of the life cycle of HBV and describes the complex regulation (including epigenetic mechanism) of the HBV cccDNA to ensure viral persistence [15]. Lack of repressive histone marks and underrepresentation of CpG islands on the viral genome ensure the rapid activation of HBV genome transcription in the infected cell. The review further discusses virus-induced direct and indirect pathways resulting in hyper- and hypomethylation of the cellular genome and its consequences such as repression of tumor suppressor genes and genome instability. Dandri points out how the elucidation of epigenetic regulation might affect HBV therapy strategies concerning eliminating the persistent cccDNA reservoir.

Similarly, epigenetic crosstalk between latent infection and cellular environment and the use of this knowledge in novel therapeutic strategies are the topic of the review by Lange, Verdikt, Ait-Ammar, and Van Lint. The authors provide a beautiful overview on HIV transcriptional regulation, latent HIV reservoir in patients, and its implication on crosstalk between proviral sequences (e.g., the high abundant defective elements) and the cellular environment [17]. The authors interpret the current knowledge on epigenetic control of HIV latency, current findings on histone modifications, DNA methylation, and long non-coding RNAs in the light of the establishment of a latent HIV reservoir and the impact of proviral activity on co-morbidities in HIV-infected patients. Knowledge of epigenetic regulation of HIV latency is used to identify latency-reversing agents, which together with antiretroviral therapy force the reversal of proviral transcriptional repression (“shock”) and subsequently eliminate the reactivated cell (“kill”). However, the efficient elimination of the latent reservoir seems to be challenging, and alternative strategies with irreversibly shutting down proviral transcription being a new therapeutic strategy are called “block” and “lock.”

Not only viruses use the host cell to ensure persistence, but also parasites and some bacteria are dependent on the cellular machinery to efficiently replicate and complete their life cycle. However, bacteria and parasites seem to possess an extended toolbox of how to manipulate the cellular epigenetic machinery. Expression of bacterial or parasitic enzymes functioning as methyltransferases or induction of histone modifications not described in mammals is two strategies of how parasites and bacteria change the epigenome of the host cell. Dong and Hamon provide an excellent overview and discussion on different mechanisms employed by bacteria (mostly intracellular bacteria such as *Mycobacteria*, *Chlamydia*, and *Legionella*) to induce histone modifications and chromatin modifications [18]. The authors illustrate the current knowledge on how LPS or another bacterial sensing of the cell triggers H3S10 phosphorylation and H3S10K14 phosphorylation/ acetylation at inflammatory genes resulting in open chromatin conformation and accessibility of the promoter by NFκB. This cascade can be successfully interrupted by bacterial effector proteins, which inhibit the mitogen-activated protein kinase (MAPK) signaling pathway at different steps. Interestingly and so far, only described for bacteria, bacteria encode for their histone methyltransferase with active SET domains that are responsible for cellular histone methylation. Furthermore, *Listeria* encoded a methyltransferase, RomA, which induces histone modifications, H3K14me3, in human cells at promoter regions of inflammatory genes, which have not been previously described. Finally, Dong and Hamon point out another strategy on how histone modification is changed during bacterial infection: low level of lipopolysaccharide (LPS) stimulation of macrophages results in epigenetic reprogramming (mostly at enhancer elements) responsible for increased basal expression of innate immunity genes responsible for an enhanced response during a second challenging event of these cells. The review by Villares, Berthelet, and Weitzman focuses on parasites of the so-called apicomplexa (a subgroup of unicellular endoparasites including *Plasmodia*, *Toxoplasma*, and *Cryptosporidia* infections in humans and *Theileria* as a known parasitic pathogen in bovine). The authors reflect examples on how these pathogens manipulate the epigenome of the host cell to ensure their persistence [19]: (1) induction of cellular methyltransferase such as SET and MYND domain-containing 3 (SMYD3) during *Theileria* infection and subsequent H3K4 methylation and transcriptional activation; (2) secretion of parasitic methyltransferases, e.g., *Toxoplasma*-secreted TEEGR (*Toxoplasma* E2F4-associated EZH2-inducing gene regulator), and subsequent enhancer of zeste 2 polycomb repressive complex 2 subunit (EZH2) expression and polycomb-mediated H3K27me3 repression; (3) secretion of parasitic-encoded ncRNAs, such as *Cryptosporidia*-encoded Cdg7_Flc_0990 transcripts and subsequent host gene promoter repression by recruiting G9a, a cellular histone methyltransferase responsible for H3K9me3 and repressive chromatin. Interestingly, *Theileria* induces host cell miRNAs, e.g., oncomiR miR-155, which results in c-Jun protein stabilization and transformation of the cell infected by the parasite. This way, *Theileria* ensures its persistence by transformation and subsequent prolonging the host cell viability. A similar mechanism in the induction of cellular miRNAs is discussed for *Toxoplasma* and *Crytosporidiae*.

In conclusion, this series of reviews addresses the complex epigenetic crosstalk between pathogens and their hosts, the consequences for the infection cycle, e.g., ensuring pathogen persistence and immune evasion. By including reviews on different viral families, bacteria, and parasites, the collection points out the similar strategies persistent pathogens apply and highlights unique features of each taxonomic kingdom. While recent work focused on how epigenetic changes on viral genomes are characteristics for specific viral transcription programs, e.g., lytic replication or latent/persistent infection, very little is known on epigenetic changes on the host genome and their contribution to cellular changes and maybe diseases. Future research is needed to (1) define the epigenetic changes on the host genome during infection, (2) define epigenetic changes during diseases associated with these pathogens, and (3) develop models to mirror the infection-induced epigenetic changes residing in (a) productive infection, (b) persistence, and (c) diseases. With this in place, it is tempting to use the gained knowledge on epigenetic control to tailor novel therapeutic approaches to eliminate persistent/latent infection and/or their associated pathogeneses.
